# Analysis of Expression Profiles of CircRNA and MiRNA in Oviduct during the Follicular and Luteal Phases of Sheep with Two Fecundity (*FecB* Gene) Genotypes

**DOI:** 10.3390/ani11102826

**Published:** 2021-09-28

**Authors:** Zhifeng Li, Xiaoyun He, Xiaosheng Zhang, Jinlong Zhang, Xiaofei Guo, Wei Sun, Mingxing Chu

**Affiliations:** 1 Key Laboratory of Animal Genetics, Breeding and Reproduction of Ministry of Agriculture and Rural Affairs, Institute of Animal Science, Chinese Academy of Agricultural Sciences, Beijing 100193, China; lizhifeng017@gmail.com (Z.L.); hedayun@sina.cn (X.H.); 2 College of Animal Science and Technology, Yangzhou University, Yangzhou 225009, China; 3 Tianjin Institute of Animal Sciences, Tianjin 300381, China; zhangxs0221@126.com (X.Z.); jlzhang1010@163.com (J.Z.); guoxfnongda@163.com (X.G.); 4 Joint International Research Laboratory of Agriculture and Agri-Product Safety, The Ministry of Education of China, Yangzhou University, Yangzhou 225009, China; 5 Jiangsu Co-Innovation Center for Important Animal Infectious Diseases and Zoonoses, Yangzhou University, Yangzhou 225009, China

**Keywords:** sheep, oviduct, fecundity, circRNA, miRNA, follicular phase, luteal phase

## Abstract

**Simple Summary:**

The oviduct is an important part of the female reproductive organs, but few people pay attention to its role in the reproductive process. In this study, we perform RNA-Seq to analyze the expression of circRNA and miRNA in the oviduct of sheep (*FecB*^BB^ and *FecB^++^*) during follicular and luteal phases. Enrichment analysis showed that the host genes of differentially expressed (DE) circRNAs were mainly enriched in the Rap1 signaling pathway, PI3K–Akt signaling pathway and neuroactive ligand–receptor interactions. Enrichment analysis showed that the target genes of DE miRNAs were mainly enriched in insulin secretion, the cAMP signaling pathway, the cGMP–PKG signaling pathway, the Rap1 signaling pathway, the TGF-β signaling pathway and other pathways related to reproduction. Our study, although not establishing direct causal relationships of the circRNA and miRNA changes, provides data for further exploring the mechanism of sheep fecundity.

**Abstract:**

CircRNA and miRNA, as classes of non-coding RNA, have been found to play pivotal roles in sheep reproduction. There are many reports of circRNA and miRNA in the ovary and uterus, but few in the oviduct. In this study, RNA-Seq was performed to analyze the expression profile of circRNA and miRNA in the oviduct during the follicular phase and luteal phase of sheep with *FecB*^BB^ and *FecB^++^* genotypes. The results showed that a total of 3223 circRNAs and 148 miRNAs were identified. A total of 15 DE circRNAs and 40 DE miRNAs were found in the comparison between the follicular phase and luteal phase, and 1 DE circRNA and 18 DE miRNAs were found in the comparison between the *FecB*^BB^ genotype and *FecB*^++^ genotype. GO and KEGG analyses showed that the host genes of DE circRNAs were mainly enriched in the Rap1 signaling pathway, PI3K–Akt signaling pathway and neuroactive ligand–receptor interactions. Novel_circ_0004065, novel_circ_0005109, novel_circ_0012086, novel_circ_0014274 and novel_circ_0001794 were found to be possibly involved in the oviductal reproduction process. GO and KEGG analyses showed that the target genes of DE miRNAs were mainly enriched in insulin secretion, the cAMP signaling pathway, the cGMP–PKG signaling pathway, the Rap1 signaling pathway and the TGF-β signaling pathway, and the target genes *LPAR1*, *LPAR2*, *FGF18*, *TACR3*, *BMP6*, *SMAD4*, *INHBB*, *SKP1* and *TGFBR2* were found to be associated with the reproductive process. Miranda software was used to identify 27 miRNAs that may bind to 13 DE circRNAs, including miR-22-3p (target to novel_circ_0004065), miR-127, miR-136 (target to novel_circ_0000417), miR-27a (target to novel_circ_0014274) and oar-miR-181a (target to novel_circ_ 0017815). The results of this study will help to elucidate the regulatory mechanisms of circRNAs and miRNAs in sheep reproduction. Our study, although not establishing direct causal relationships of the circRNA and miRNA changes, enriches the sheep circRNA and miRNA database and provides a basis for further studies on sheep reproduction.

## 1. Introduction

Small Tail Han (STH) sheep are widely bred in China for their year-round estrus and polyembryony [1]. The lambing rate of primiparous ewes is about 200%, and for non-primiparous ewes it is higher than 250% [2]. Studies have demonstrated the vital role of the *FecB* (*BMPR1B*) gene in sheep fecundity [3,4]. Our previous research found that all three genotypes of *FecB* (*FecB*^BB^, *FecB*^B+^ and *FecB*^++^) are distributed in STH sheep, and there is a significant correlation between three genotypes of *FecB* and the litter size of ewes [5]. Thus, we regard STH sheep as a suitable animal model to study the molecular mechanism of *FecB* gene regulation of reproductive traits.

The process of the reproduction of sheep is complicated, in which ovarian follicle development, ovulation and luteinization occur. Most studies of fecundity have focused on the ovaries and uterus [6,7], and little is known about the oviduct, a key tissue in sheep reproduction. In mammals, the oviduct is the first site of maternal contact with the embryo. This contact occurs in the first four days after fertilization [8]. In order to ensure an optimal environment during implantation, the oviduct needs to exchange information with the embryo [9]. In this way, a molecular mechanism through which the oviduct interacts with the developing embryo is initiated, and the body can successfully perform epigenetic reprogramming and activate the embryonic genome [10,11]. This embryo–oviduct interaction may cause changes in transcription, which may have effects on offspring and lead to changes in fecundity. In addition, mammalian oviduct epidermal cells can synthesize and secrete a range of proteins and affect embryonic development through a variety of signaling pathways, all of which highlight the role of the oviduct in sheep’s reproductive process. Therefore, further understanding the molecular regulatory mechanisms and signaling pathways of oviduct-related functions is important for studying the reproductive characteristics of sheep.

Circular RNAs are a new type of non-coding RNA with regulatory functions [12,13]. They have a closed loop structure and are abundant in the eukaryotic transcriptome. Most circRNAs are composed of exon sequences, which are conserved in the transcriptome and have tissue specificity and expression specificity at different developmental stages. Studies have found that circRNA can act as a sponge for miRNA, which leads to the inhibition of miRNA activity and increased levels of target genes [14]. For example, circRNA-9119 affects endometrial receptivity in dairy goats by acting as a sponge for microRNAs that bind to miR-26a, preventing it from participating in the mediation process [15]. The further development of high-throughput sequencing has brought research on circRNAs to the molecular level, and more and more reports on circRNAs in sheep have been found in the pituitary [16], hypothalamus [17], uterus [18] and other tissues.

MicroRNAs are a class of non-coding single-stranded RNA molecules with a length of approximately 22 nucleotides, encoded by endogenous genes. They are involved in the regulation of post-transcriptional gene expression in mammals. MicroRNAs are initially pri-miRNAs, which are processed to become precursors of microRNAs of about 70 to 90 bases in length. Pre-miRNAs are processed by enzymes to become mature miRNAs. Some microRNAs are important regulators involved in the ovarian follicular and luteal development [19]. For example, miR-224, miR-378 and miR-383 were found to regulate aromatase expression during follicle development, and miR-17-5p and let-7b were vital for the development of the luteum [20]. MicroRNAs also play key roles in growth and development, such as muscle growth [21] and neurodevelopment [22].

To explore the roles of circRNAs and miRNAs in the oviduct between STH sheep with *FecB*^BB^ (MM sheep) and STH sheep with *FecB*^++^ (WW sheep), RNA-Seq was performed and comparison of the expression profiles of circRNA and miRNA of MM sheep and WW sheep was conducted. In addition, GO and KEGG enrichment analyses were performed on the host genes of DE circRNAs and predicted target genes of DE miRNAs. This may help us to better understand the molecular mechanisms of circRNAs and miRNAs during oviductal reproduction in sheep.

## 2. Materials and Methods

### 2.1. Animal Processing and Sample Acquirement

All sheep involved in this experiment were approved by the Science Research Department (in charge of animal welfare issues) of IAS-CAAS and ethical approval was given by the Animal Ethics Committee of the IAS-CAAS (No. IAS 2019-49). All sheep were from Yuncheng Breeding Sheep Farm (Yuncheng County, China), where they all obtained similar feeding conditions.

First, 890 Small Tail Han (STH) sheep were genotyped in pre-experiment. Then, six sheep with *FecB*^BB^ genotype and six sheep with *FecB*^++^ genotype were selected according to average age, average weight, body length and chest circumference.

Then, all the selected STH sheep were treated with controlled internal drug releasing (CIDR, progesterone 300 mg) to achieve synchronous estrus. Three *FecB*^BB^ sheep and three *FecB*^++^ sheep were euthanized in the follicular phase and the rest in the luteal phase. The oviductal tissues were collected and stored at −80 °C for RNA extraction. Details on phenotypic identification, synchronous estrus treatment and period determination (follicular and luteal phase) of experimental sheep can be found in Zhang et al. [23].

The oviductal tissues of *FecB*^BB^ (mutant type, MM) sheep at follicular phase and luteal phase were named MF (*n* = 3) and ML (*n* = 3), and the oviductal tissues of the *FecB*^++^ (wild type, WW) sheep at the follicular phase and luteal phase were named WF (*n* = 3) and WL (*n* = 3), respectively.

### 2.2. RNA Extraction, Library Construction and RNA-Seq

Total RNA was extracted from the oviductal tissues of 12 sheep. TRIzol reagent (Invitrogen, Carlsbad, CA, USA) was used for total RNA extraction, according to the manufacturer’s instructions. To obtain high-quality RNA, 1% agarose electrophoresis and an Agilent 2100 Bioanalyzer (Agilent Technologies, Santa Clara, CA, USA) were used to examine the integrity and concentration of the extracted RNA. The purity of isolated RNA was also ensured using an Agilent RNA 6000 Nano Kit (Agilent Technologies).

The RNA library was constructed with 3 µg of high-quality RNA using the NEBNext^®^ Ultra™ RNA Library Prep Kit for Illumina^®^ (NEB, Ipswich, MA, USA) according to the manufacturer’s recommendations. During this process, Ribo-Zero™ GoldKits was used to remove rRNA. CircRNAs were randomly fragmented and reverse transcribed into cDNA with random primers. Second-strand cDNA was synthesized using DNA polymerase I, RNase H, dNTPs and buffer, and the cDNA fragments were purified by QiaQuick PCR, repaired at the end, tagged was a poly(A) and ligated into Illumina sequencing adapters.

The fragments with lengths of 18–30 nt, which were obtained from total RNA by the gel separation technique, were used as templates to synthesize the first strand of complementary DNA (cDNA). The second strand of cDNA was also synthesized in the presence of deoxynucleoside triphosphates (dNTPs), ribonuclease H and DNA polymerase I. Then, the obtained double-stranded cDNA was processed with end-repair, the addition of base A and sequencing adaptors and uracil-N-glycosylase (UNG) enzyme digestion. Finally, polymerase chain reaction was conducted to build the miRNA library.

cDNA and miRNA libraries were then sequenced using a HiSeq X-Ten platform, and all the sequencing was conducted in Novogene Bioinformatics Technology Co. Ltd. (Beijing, China). Raw data of the RNA-Seq have been deposited in the SRA database (Accession number: PRJNA658731).

### 2.3. Identification and Differential Expression Analysis of ciriRNA

Raw reads were first filtered and low quality reads, reads containing adapter and reads containing ploy-N were removed to obtain clean reads. Clean reads of circRNAs were mapped to the reference genome (Oar_v3.1) by the HiSAT2 alignment method. CIRI is an efficient and fast tool to identify circRNAs [24]. The BWA-MEM algorithm was used to conduct a sequence splitting comparison to ensure the reliability of other circRNAs, and then the SAM file was scanned to find PCC (paired chiastic clipping) and PEM (paired-end mapping) sites, and GT-AG splicing signals [25]. Moreover, we used dynamic programming algorithm to re-align the sequence with the junction site. CircRNAs were blasted against the circBase for annotation. The circRNAs that could not be annotated were defined as novel circRNAs. Statistical analysis was performed on the identified circRNA types, chromosome distribution and length distribution.

Relative expression of circRNAs was analyzed by TPM (transcripts per million reads) [26]. The DEseq2 package was used to identify DE circRNAs across groups [27]. We identified circRNAs with a |fold change| of >1.5 and a *p*-value of <0.05 between two groups as DE. In addition, miRanda v3.3a was used to predict the miRNA binding site of circRNAs.

### 2.4. Identification and Differential Expression Analysis of miRNA

Several criteria were conducted to obtain clean miRNA reads, including removing reads without a 3′ adapter, reads without insert fragment, reads with lengths beyond the normal range, raw reads containing poly A/T and some low-quality reads using in-house scripts. The clean data of miRNAs were mapped to miRBase to identify known miRNAs. Then, the remaining clean reads that were not mapped to sheep miRBase were mapped to sheep reference genome (Oar_3.1) to predict novel miRNAs by miRDeep 2.0.0.8. Relative expression of miRNAs was analyzed by TPM. The DEseq2 package, which was based on negative binomial distribution, was used to identify DE miRNAs across groups. Additionally, the thresholds of |fold change| > 1.5 and *p*-value < 0.05 were set to identify DE miRNAs. In addition, miRanda v3.3a, PITA and RNAhybrid v2.1.2 were used to predict the target genes of miRNAs.

### 2.5. GO and KEGG Analyses of Host Genes of DE circRNAs and Predicted Target Genes of DE miRNAs

According to the correspondence between circRNAs and their host genes, Gene Ontology (GO) and Kyoto Encyclopedia of Genes and Genomes (KEGG) enrichment analysis were performed for each group of host genes with DE circRNAs. Additionally, GO and KEGG enrichment analyses were performed for each group of predicted target genes with DE miRNAs. The GOseq R package was used to analyze GO enrichment of host genes of DE circRNAs and predicted target genes of DE miRNAs. KEGG annotations on host genes of DE circRNAs and predicted target genes of DE miRNAs were also conducted based on KEGG database (http://www.genome.jp (accessed on 1 June 2019)). The hypergeometric test method was applied to assess significantly enriched GO terms and KEGG pathways, and those with *p* < 0.05 were thought to be significantly enriched.

### 2.6. Validation of the Expression of circRNAs and miRNAs

To further confirm the circRNA and miRNA sequencing data, four DE circRNAs and five DE miRNAs were selected randomly. We designed forward and reverse primers encompassing circRNA-specific, back-splice junctions for each candidate circRNA (Table 1). The reverse transcription of circRNA was performed using PrimeScript™ RT reagent kit (TaKaRa, Dalian, China). Then, RT-qPCR was performed using SYBR Green Real-time PCR Master Mix (TOYOBOCO, LTD, Osaka, Japan) in a Roche LightCycler 480II (Roche, Basel, Sweden), according to the manufacturer’s instructions.

The reverse primers of miRNAs were designed using tailing reaction, which increases the accuracy and specificity of detection (Table 1). The forward primer is included in miRcute Plus miRNA qPCR Kit (SYBR Green). Afterwards, the reverse transcription of miRNA was performed using miRcute Plus miRNA First-Strand cDNA Kit (TIANGEN, Beijing, China), followed by use of miRcute Plus miRNA qPCR Kit (SYBR Green) (TIANGEN, Beijing, China) to conduct RT-qPCR through the Roche Light Cycler^®^ 480II.

Real-time PCR was performed at 95 °C for 10 min, followed by 45 cycles of 95 °C for 15 s, 60 °C for 60 s and 72 °C for 30 s. The β-Actin and U6 small nuclear RNA were used as internal control to normalize target gene expression, respectively. The results obtained from RT-qPCR were calculated using the 2^−∆∆Ct^ method [28] and then processed by SPSS 22.0. Finally, PCR products were gel extracted and subjected to Sanger sequencing.

## 3. Results

### 3.1. Overview of circRNA Profiles of Small Tail Han Sheep Oviduct

**Figure 1:**
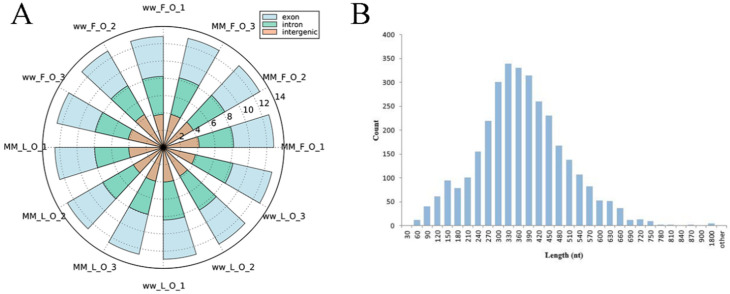
General characteristics of circRNAs in the sheep oviduct. (**A**) Source of circRNAs in genome regions. (**B**) Distribution of length of spliced circRNAs (the count represents the number of circRNAs).

**Figure 2:**
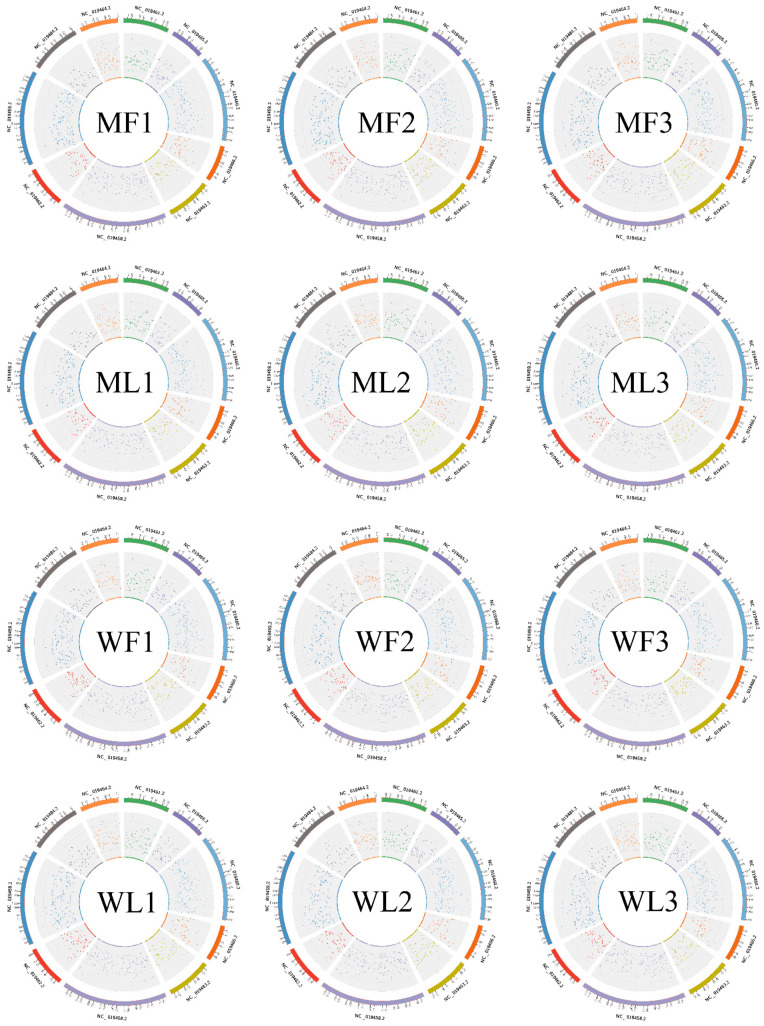
The density statistics of circRNA of each chromosome between MF, ML, WF and WL groups. The figures next to the circles are chromosome IDs.

To identify circRNAs in MF, ML, WF and WL groups of Small Tail Han sheep, the cDNA library was constructed. All samples were divided into four groups with three replications, including MF (MM_F_O_1, MM_F_O_2, MM_F_O_3), ML (MM_L_O_1, MM_L_O_2, MM_L_O_3), WF (ww_F_O_1, ww_F_O_2, ww_F_O_3) and WL (ww_L_O_1, ww_L_O_2, ww_L_O_3). The number of raw reads (on average) in the four groups was 93,948,897 (MF), 93,872,202 (ML), 94,768,814 (WF) and 99,720,231 (WL), respectively. The number of clean reads (on average) was 91,719,898 (MF), 90,158,838 (ML), 91,784,720 (WF) and 96,577,435 (WL), respectively. The average rate aligned to the Ovis aries genome was 89% (Table 2). A total of 3223 circRNAs were identified after mapping to the reference sequence (Table S1). CircRNAs are mainly derived from spliced exons, followed by introns, and least from intergenic regions (Figure 1A). The length distribution of the spliced circRNAs was mainly between 200 bp and 500 bp (Figure 1B). The circRNA density statistics for each chromosome revealed that circRNAs were distributed on chromosomes 1 to X (Table S1). Figure 2 shows that the highest densities were on chromosomes 1 to 9 and chromosome X. Most circRNAs were located on chromosomes 1, 2 and 3.

### 3.2. Overview of miRNA Profiles of Small Tail Han Sheep Oviduct

**Figure 3:**
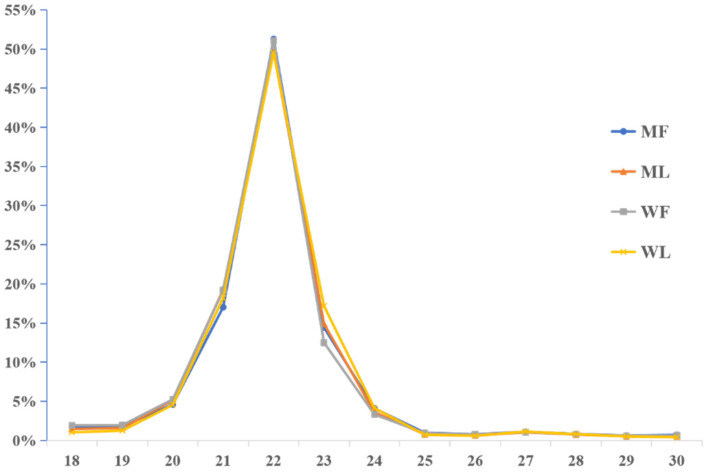
Sequence length distribution of sRNAs.

**Figure 4:**
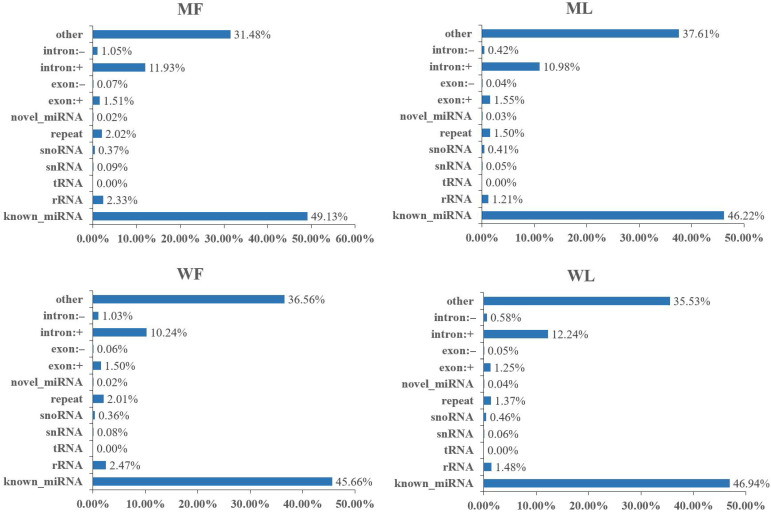
The category of identified RNAs after miRNA sequencing. Other refers to the proportion of sRNAs not aligned to the other categories in the diagram.

Meanwhile, an miRNA library was constructed to identify miRNAs in MF, ML, WF and WL groups of Small Tail Han sheep. The number of raw reads (on average) in four groups was 13,310,733 (MF), 11,631,773 (ML), 12,325,568 (WF) and 11,798,850 (WL), respectively. The number of screened reads (on average) was 12,097,257 (MF), 10,817,158 (ML), 10,964,721 (WF) and 11,021,986 (WL), respectively. The average rate aligned to the Ovis aries genome was 90.56% (Table 3). The sequence length distribution of sRNAs was listed, with 21-22nt miRNAs accounting for the majority (Figure 3). We classified the identified RNAs after sequencing, with more than 45% of known miRNAs and less than 1% of novel miRNAs (Figure 4). A total of 148 known miRNAs were obtained, and 23 novel miRNAs were obtained by miRDeep prediction (Table S2).

### 3.3. Differential Expression Analysis of circRNAs

**Figure 5:**
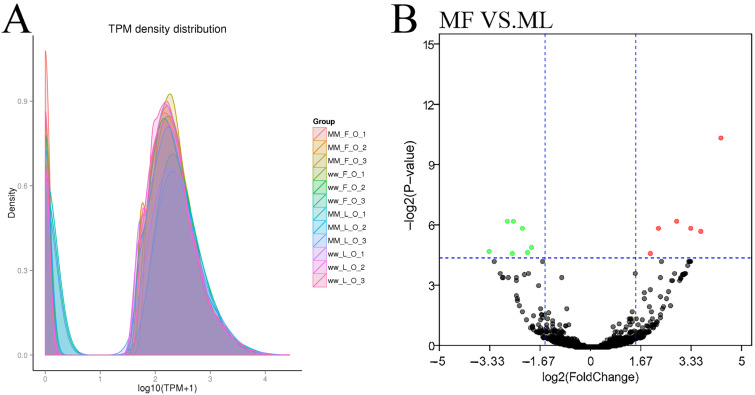
The information of circRNAs. (**A**) The TPM density distribution of circRNA. The expression was normalized with TPM (Transcripts per million). (**B**) Differentially expressed circRNAs in MF vs. ML. The abscissa represents the change in circRNA expression multiple in different experimental groups. The ordinate represents the statistically significant degree of circRNA. The dots represent circRNAs. The black dots represent the circRNAs with no significant difference, the red dots represent the significantly up-regulated circRNAs and the green dots represent the significantly down-regulated circRNAs.

The known and novel circRNAs’ expression levels were calculated for each sample, and the expression levels were normalized by TPM (Figure 5A). DE circRNAs were identified according to the condition of |fold change| > 1.5 and *p*-value < 0.05. In the comparisons between the follicular phase (F) and luteal phase (L), a total of 15 (seven up-regulated and eight down-regulated) and no DE circRNAs were revealed between MF vs. ML (Figure 5B, Table S3) and WF vs. WL, respectively.

In the comparisons between the *FecB*^BB^ genotype (M) and *FecB*^++^ genotype (W), a total of one (up-regulated) and no DE circRNAs were revealed between MF vs. WF (Table S3) and ML vs. WL, respectively.

### 3.4. Differential Expression Analysis of miRNAs

**Figure 6:**
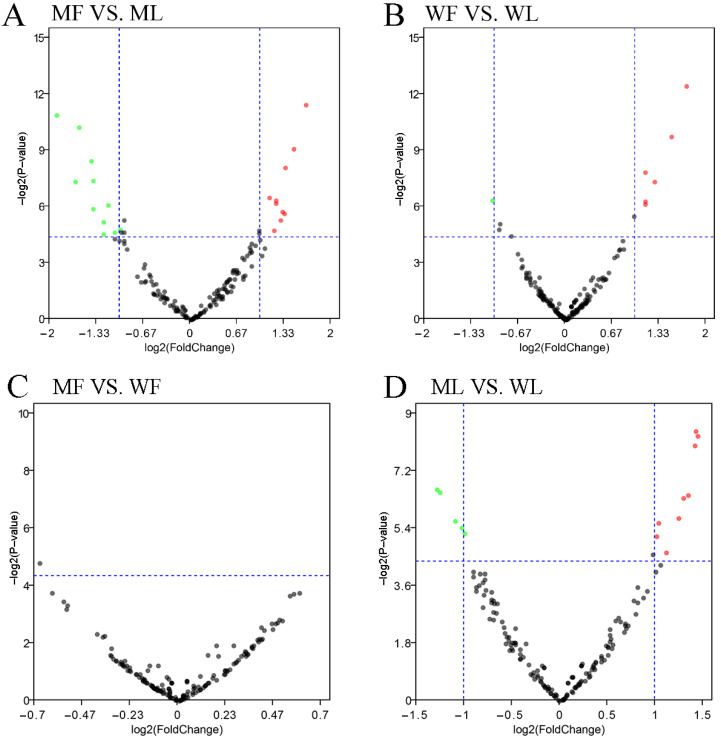
Analysis of differentially expressed miRNAs. (**A**) Differentially expressed miRNAs in MF vs. ML. (**B**) Differentially expressed miRNAs in WF vs. WL. (**C**) Differentially expressed miRNAs in MF vs. WF. (**D**) Differentially expressed miRNAs in ML vs. WL. The abscissa represents the change in miRNA expression multiple in different experimental groups. The ordinate represents the statistically significant degree of miRNA. The dots represent miRNAs. The black dots represent the miRNAs with no significant difference, the red dots represent the significantly up-regulated miRNAs and the green dots represent the significantly down-regulated miRNAs.

A total of 171 miRNAs were identified in the oviduct of Small Tail Han sheep in four groups, including 23 novel miRNAs and 148 known miRNAs. DE miRNAs were identified according to the condition of |fold change| > 1.5 and *p*-value < 0.05. In the comparisons between the follicular phase (F) and luteal phase (L), a total of 29 (12 up-regulated and 17 down-regulated) and 11 (seven up-regulated and four down-regulated) DE miRNAs were revealed between MF vs. ML (Figure 6A, Table S4) and WF vs. WL (Figure 6B, Table S4), respectively.

In the comparisons between the *FecB*^BB^ genotype (M) and *FecB*^++^ genotype (W), a total of two (one up-regulated and one down-regulated) and 16 (10 up-regulated and 6 down-regulated) DE miRNAs were revealed between MF vs. WF (Figure 6C, Table S4) and ML vs. WL (Figure 6D, Table S4), respectively. In order to analyze the clustering model of differentially expressed miRNAs among the four groups, 42 DE miRNAs were clustered using K-means and SOM cluster analysis (Figure S1).

**Figure 7:**
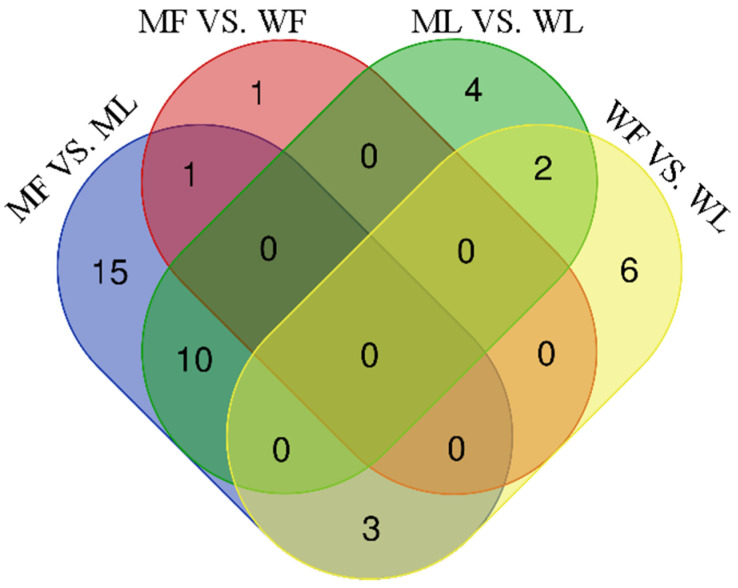
The Venn diagrams of miRNA of different comparison groups. The figures represent the number of miRNAs contained in each group, and the overlap represents the number of the common miRNAs.

In addition, the Venn diagrams for each group comparison showed that three miRNAs were in common in MF vs. ML and WF vs. WL, while no common miRNAs were found in MF vs. WF and ML vs. WL (Figure 7).

### 3.5. GO and KEGG Pathway Enrichment Analyses of circRNAs

**Figure 8:**
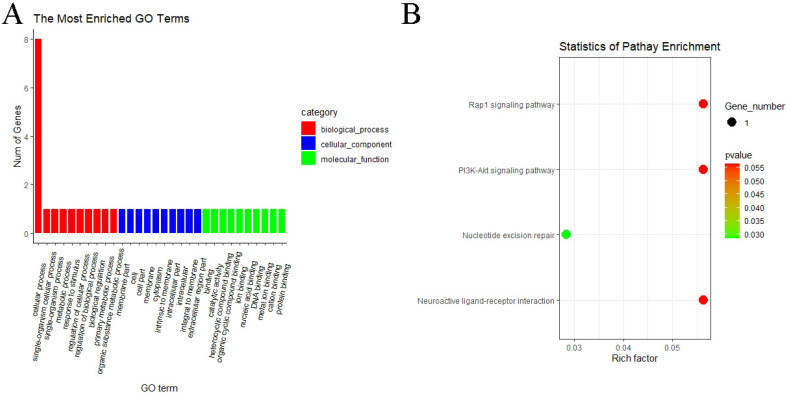
GO and KEGG analyses of DE circRNA host genes. (**A**) GO analysis of DE circRNA host genes of MF vs. ML. The longitudinal and horizontal axes represent the number of GO terms and names, respectively. (**B**) KEGG analysis of DE circRNA host genes of MF vs. ML. The longitudinal and horizontal axes represent the enrichment pathways and rich factor of these pathways, respectively. The spot size represents the number of differentially expressed genes enriched in each pathway, and the color of the spot represents the *p*-value of each pathway.

GO and KEGG pathway analyses were performed to know more about the features of DE circRNAs. In the MF vs. ML group, GO analyses of host genes revealed significantly enriched terms in biological process (BP), molecular function (MF) and cellular components (CC). The result showed that host genes with DE circRNAs were annotated into 366 functional subclasses, and 106 GO items were significant. A total of 88, 17 and 1 GO terms were significantly enriched in BP, MF and CC. Among them, we found that many host genes with DE circRNAs were enriched in cellular process (GO:0009987), membrane part (GO:0044425), binding (GO:0005488), catalytic activity (GO:0003824) and other subclasses (Figure 8A). We also analyzed the KEGG pathway of host genes of DE circRNAs, and the result indicated that the RAP1 signaling pathway (oas04015), PI3K–Akt signaling pathway (oas04151), nucleotide excision repair (oas03420) and neuroactive ligand–preceptor interaction (oas04080) were enriched in MF vs. ML (Figure 8B). These above pathways may be involved in the regulation of oviductal function in sheep. GO and KEGG analyses were not performed for the remaining three groups since the number of DE circRNAs was too small.

### 3.6. GO and KEGG Pathway Enrichment Analyses of miRNAs

**Figure 9:**
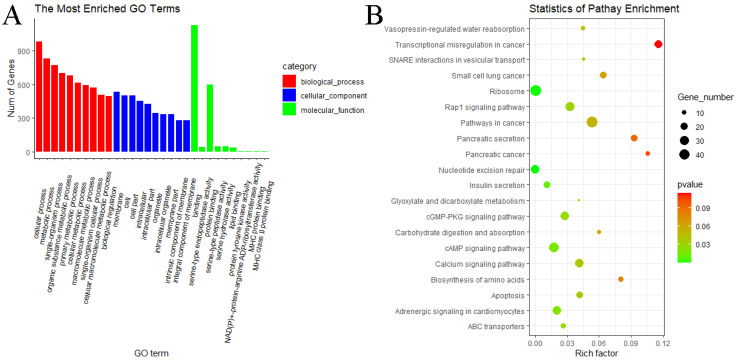
GO and KEGG analyses of predicted target genes of DE miRNAs in MF vs. ML. (**A**) GO analysis of predicted target genes of DE miRNAs in MF vs. ML. (**B**) KEGG analysis of predicted target genes of DE miRNAs in MF vs. ML.

In the MF vs. ML group, GO analyses of predicted target genes of DE miRNAs revealed significantly enriched terms in biological process (BP), molecular function (MF) and cellular components (CC). The result showed that predicted target genes with DE miRNAs were annotated into 3068 functional subclasses, and 209 GO items were significant. A total of 117, 68 and 24 GO terms were significantly enriched in BP, MF and CC. Among them, we found that many predicted target genes with DE miRNAs were enriched in cellular process (GO:0009987), metabolic process (GO:0008152), binding (GO:0005488), cell (GO:0005623) and other subclasses (Figure 9A). KEGG analysis of the predicted target genes of DE miRNAs showed that predicted target genes were significantly enriched in 13 pathways, including ribosome (oas03010), nucleotide excision repair (oas03420), insulin secretion (oas04911), cAMP signaling pathway (oas04024), cGMP–PKG signaling pathway (oas04022) and Rap1 signaling pathway (oas04015) (Figure 9B).

**Figure 10:**
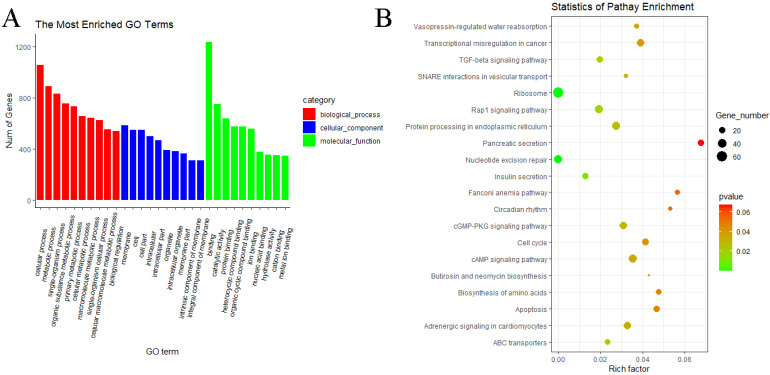
GO and KEGG analyses of predicted target genes of DE miRNAs in WF vs. WL. (**A**) GO analysis of predicted target genes of DE miRNAs in WF vs. WL. (**B**) KEGG analysis of predicted target genes of DE miRNAs in WF vs. WL.

In the WF vs. WL group, the result showed that predicted target genes with DE miRNAs were annotated into 3075 functional subclasses, and 160 GO items were significant. A total of 80, 61 and 19 GO terms were significantly enriched in BP, MF and CC. Among them, we found that many predicted target genes with DE miRNAs were enriched in cellular process (GO:0009987), metabolic process (GO:0008152), binding (GO:0005488), catalytic activity (GO:0003824), membrane (GO:0016020) and other subclasses (Figure 10A). KEGG analysis of the predicted target genes of DE miRNAs showed that predicted target genes were significantly enriched in 17 pathways, including ribosome (oas03010), nucleotide excision repair (oas03420), TGF-beta signaling pathway (oas04350), insulin secretion (oas04911), cAMP signaling pathway (oas04024), cGMP–PKG signaling pathway (oas04022) and Rap1 signaling pathway (oas04015) (Figure 10B).

GO and KEGG analyses were not performed for the comparisons between the *FecB*^BB^ genotype and *FecB*^++^ genotype since the number of DE miRNAs was too small.

### 3.7. Regulatory Networks of miRNAs and circRNAs

**Figure 11:**
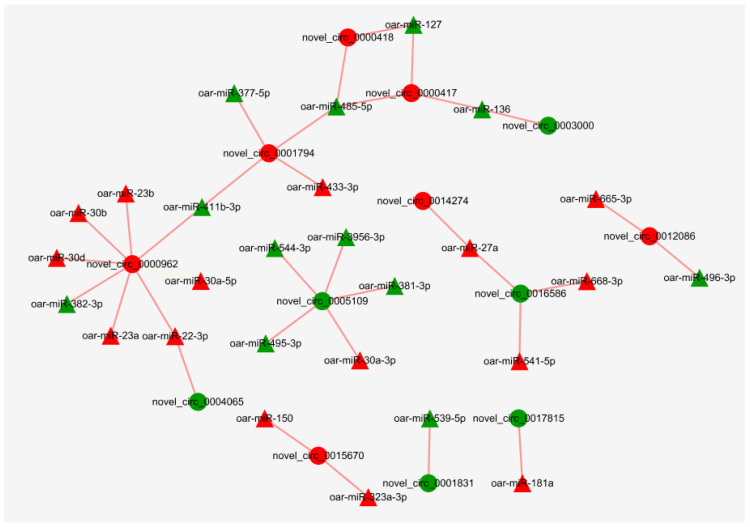
Regulatory networks of miRNAs–circRNAs in MF vs. ML. Triangular nodes represent miRNA, and circular nodes represent circRNA. Red represents up-regulated expression, whereas green represents down-regulated expression.

MiRanda software was used to predict the binding sites of miRNAs to circRNAs. The results showed that 153 miRNAs may bind to 2824 circRNAs. There were multiple miRNA binding sites on the same circRNA, and the same miRNA also targeted more than one circRNA. Since DE circRNAs are only present in the MF vs. ML group, network diagrams were made only for this group. After screening, 27 miRNAs were found to potentially bind to 13 DE circRNAs in MF vs. ML (Figure 11, Table S5). It was found that oar-miR-181a, oar-miR-136/oar-miR-127, oar-miR-22-3p and oar-miR-27a related to sheep reproduction were correspondingly correlated with novel_circ_0017815 (RBPMS), novel_circ_0000417 (LOC106990833), novel_circ_0004065 (PAWR) and novel_circ_0016586 (INPP5F).

### 3.8. Validation of circRNA Expression

**Figure 12:**
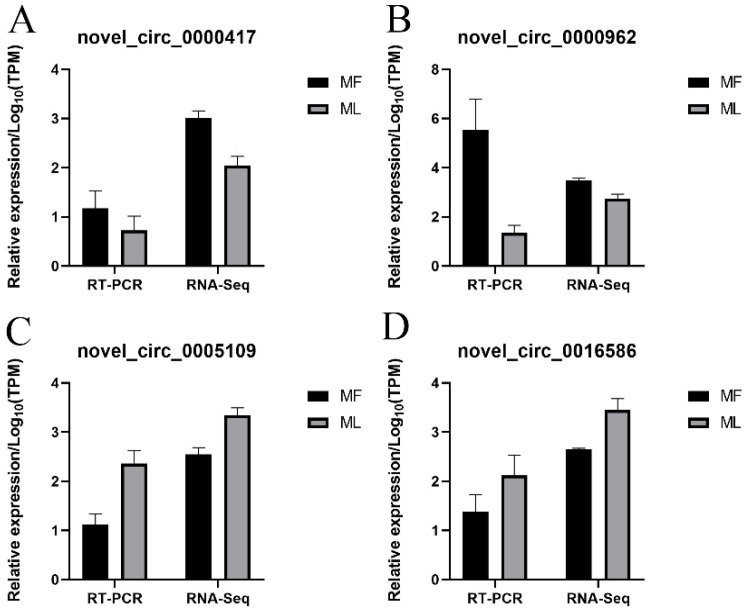
Validation of circRNAs expression by RT-qPCR. Validation results of four selected circRNAs in MF and ML by RT-qPCR (**A**–**D**).

RT-qPCR was conducted to confirm the sequencing data of circRNAs. Our results indicated that the four selected circRNAs showed similar expression trends to the sequencing data, suggesting the reliability of our sequencing results (Figure 12).

### 3.9. Validation of miRNA Expression

**Figure 13:**
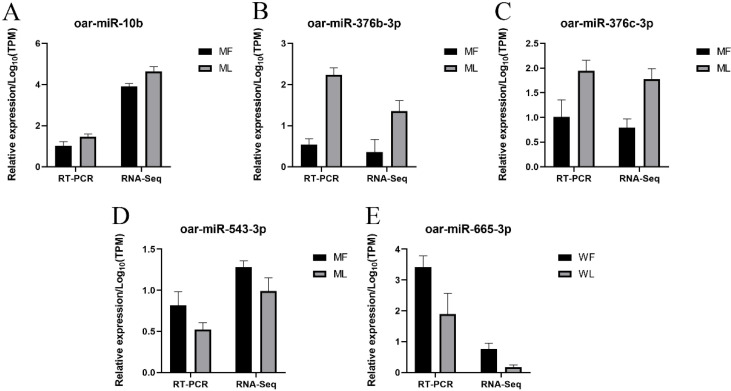
Validation of miRNAs expression by RT-qPCR. Validation results of four selected miRNAs in MF and ML by RT-qPCR (**A**–**D**); Validation results of one selected miRNA in WF and WL by RT-qPCR (**E**).

RT-qPCR was conducted to confirm the sequencing data of miRNAs. Our results indicated that the five selected miRNAs showed similar expression trends to the sequencing data, suggesting the reliability of our sequencing results (Figure 13).

## 4. Discussion

At present, the research on sheep’s fecundity is mainly about several major genes such as *BMPR1B*, *BMP15* and *GDF9*, and more novel biomarkers of fecundity need to be identified. It has been previously reported that scholars conducted transcriptomic analysis of the hypothalamus [17], uterus [29] and ovary [30], etc., that related to prolificacy. However, little transcriptomic research has been performed on the oviduct, as a key part of the female reproductive system. Recently, studies have shown that the oviduct plays an important role in the fertilization and pre-implantation development of the embryo [9,31,32]. Thus, in this study, STH sheep with excellent lambing performance (year-round estrous, 2.61 lambs per year on average) were selected, and the oviducts were subjected to RNA-Seq to study the transcriptome profiles of sheep with two *FecB* genotypes during the follicular and luteal phases.

### 4.1. Transcriptomic Profiles

In this study, we identified 3223 circRNAs and 148 miRNAs in the sheep oviduct. We also analyzed the distribution of circRNAs in the genome regions and the length distribution of spliced circRNAs. Most of the circRNA genome composition in sheep uterus was introns [33], while the majority in the oviduct was composed of exons. The length distribution of the spliced circRNAs was mainly between 200 bp and 500 bp, which was inconsistent with the results from the sheep uterus but similar to the results from sheep mammary glands [34]. The length distribution of miRNAs in the pituitary and ovary of sheep was similar to our results [35,36], with 22 nt miRNAs accounting for the majority. Thus, circRNAs may be tissue-specific and miRNAs are conservative in different tissues.

A total of 15 DE circRNAs and 40 DE miRNAs were identified in the comparisons between the follicular phase and luteal phase, and 1 DE circRNA and 18 DE miRNAs were identified in the comparisons between the *FecB*^BB^ genotype and *FecB*^++^ genotype. The results showed that more DE circRNAs and DE miRNAs were obtained in the comparisons between the follicular phase and luteal phase, but fewer in the comparisons between the *FecB*^BB^ genotype and *FecB*^++^ genotype, indicating that different *FecB* genotypes have less impact on the oviductal transcriptome.

### 4.2. Differential Expression Analysis and Functional Analysis of circRNAs

Fifteen DE circRNAs were identified in MF vs. ML, but no DE circRNAs were found in WF vs. WL. It is hypothesized that DE circRNAs in MM sheep during the follicular to luteal phase may be involved in the regulation of oviductal development. Among all these DE circRNAs, the top two with the highest expression levels were novel_circ_0004065 and novel_circ_0005109, whose host genes were *PAWR* and *SMC6*, respectively. Studies have found that pro-apoptotic WT1 regulator (*PAWR*) conducted cell apoptosis, which inhibited the growth of prostate cancer cells [37]. Additionally, *PAWR* regulated the apoptosis of follicles in the rat ovary but was suppressed by FSH through the activation of the PKCζ-dependent anti-apoptotic pathway [38]. However, the expression of *PAWR* was observed up-regulated in granulosa cells (GCs), indicating the increased susceptibility of GCs to undergo apoptosis [39]. In this study, the expression level of novel_circ_0004065 was lower at the follicular phase and increased at the luteal phase, which may be explained by these studies, implying the ovary and oviduct are coordinated with each other and stay in sync during the estrous cycle [40]. Studies have shown that the structural maintenance of chromosome 6 (*SMC6*) is essential for DNA repair and the maintenance of genomic integrity [41]. Moreover, *SMC6* plays a key role in spermatogenesis and oocyte meiosis [42,43], which indicated that *SMC6* may maintain the genomic integrity of the sperm and embryo to ensure fertility. In summary, novel_circ_0004065 and novel_circ_0005109 may have important roles in the reproductive process of the oviduct and early embryonic development in sheep, and the specific mechanisms need to be further investigated.

In the comparisons between the follicular phase and luteal phase, GO and KEGG analyses were performed. The most significantly enriched circRNAs of GO terms in MF vs. ML were novel_circ_0012086 and novel_circ_0014274, whose host genes were *XPR1* and *SLC7A11.* Xenotropic and polytropic retrovirus receptor 1 (*XPR1*) is a gene encoding cellular inorganic phosphate export protein, and its mutation can cause primary familial brain calcification. The normal development of the fetus is inseparable from phosphorus. This nutrient is mainly transported from the maternal blood to the fetus via the placenta. Xu et al. [44] found that *XPR1* was highly expressed in the murine placenta, but the placenta of the murine that knocks out this gene was severely calcified. Soluble carrier family 7 member 11 (*SLC7A11*) gene is a target of p53-mediated transcriptional repression, and p53 can inhibit the uptake of cystine by repressing the expression of *SLC7A11*. Studies on mutant mice revealed that p53 plays an important role in embryonic development [45]. In addition, *SLC7A11* exists in the sperm of stallions and regulates the oxidation–reduction status of sperm by exchanging extracellular cystine (Cyss) for intracellular glutamate [46].

In KEGG pathway analysis, the host gene lysophosphatidic acid receptor 3 (*LPAR3*), whose circRNA was novel_circ_0001794, was involved in the Rap1 signaling pathway, PI3K–Akt signaling pathway and neuroactive ligand–receptor interactions. Rap1 combined with GTP activates the PI3K–Ark signaling pathway, and the PI3K–Ark signaling pathway is widely involved in various important processes of mammalian ovarian development [47], and is related to the survival and activation of primitive follicles [48], hormone secretion and so on. In addition, neuroactive ligand–preceptor interaction is related to the effect of GnRH and GnRHR. *LPAR3* was found to be expressed in mouse oviduct, placenta and uterus, and its essential role in the female reproductive system was reported [49]. Studies have found that progesterone is likely to have a direct effect on *LPAR3*, and progesterone treatment can increase the expression of *LPAR3* mRNA in the endometrium [50]. In addition, dynamic changes that occur in the organization of luminal and glandular epithelia in the endometrium during the estrous cycle are necessary to modulate the appropriate environment for the developing embryo and to allow the implantation of the conceptus [51]. The differentiation of oviductal epithelial cells is also affected by progesterone. Given the key role of progesterone, we suppose that *LPAR3* may play crucial roles in sheep reproduction. Therefore, novel_circ_0012086, novel_circ_0014274 and novel_circ_0001794, whose host genes were *XPR1*, *SLC7A11* and *LPAR3*, respectively, may have key roles in reproduction, which requires further validation.

### 4.3. Differential Expression Analysis and Functional Analysis of miRNAs

Three DE miRNAs (miR-665-3p, miR-370-3p, miR-19b) were found in both MF vs. ML and WF vs. WL, while no common miRNAs were found in MF vs. WF and ML vs. WL. This implies that these three miRNAs have an important role in the sheep oviductal reproduction process. In the comparisons between the follicular phase and luteal phase, GO and KEGG analyses were performed on the predicted target genes of miRNAs. GO terms such as cellular processes, metabolic processes and binding involved in MF vs. ML imply a high level of cellular activities between the follicular phase and luteal phase. In KEGG pathway analysis, target genes of miRNAs in MF vs. ML were found to be mainly involved in insulin secretion, the cAMP signaling pathway, the cGMP–PKG signaling pathway, the Rap1 signaling pathway and the calcium signaling pathway. Notably, *LPAR1*, *LPAR2*, *FGF18* and *TACR3* were enriched in the above pathways. Same as *LPAR3*, *LPAR1* and *LPAR2* play key roles in the female reproductive system. Studies have found that LPA medium can improve the survival and development potential of follicles, and can stimulate the cell function and E2 synthesis of mouse ovarian tissue [52]. In addition, the oviduct is an important part where gamete transport and fertilization happened. LPA was found to be involved in gamete transport, fertilization and cell signal transmission between oviductal tissue and the cumulus oocyte complex [53,54]. *LPAR2* was found to be abundantly expressed in the oviduct of cattle, suggesting that the oviduct is an important target of LPA [55]. Fibroblast growth factor 18 (*FGF18*) inhibits the secretion of estradiol and progesterone, and is a candidate factor that regulates steroidogenesis during ovarian development [56]. Moreover, *FGF18* is likely to cause granulosa cell apoptosis, thereby affecting follicular atresia [57,58]. Tachykinin receptor 3 (*TACR3*) plays a key role in regulating gonadotropin secretion and sex hormone feedback regulation of the reproductive axis [59]. *TACR3* may also be related to the regulation of granulosa cell function and changes in ovarian function [60]. In addition, the expression of *TACR3*/*TAC3* can promote the secretion of GnRH [61], which may affect sheep reproduction. Thus, these genes are likely to participate in the reproductive process of MM sheep.

In WF vs. WL, the GO terms that were involved were similar to MM sheep. In KEGG pathway analysis, the target genes of miRNAs in WF vs. WL were found to be mainly involved in the TGF-β signaling pathway, insulin secretion, protein processing in the endoplasmic reticulum, the cGMP–PKG signaling pathway and the Rap1 signaling pathway. In addition to the target genes above (*LPAR1*, *LPAR2*, *FGF18*, *TACR3*), we found that miR-370-3p and its target genes (*BMP6*, *SMAD4*, *INHBB*), miR-431 and its target gene (*SKP1*) and miR-541-5p and its target gene (*TGFBR2*) were also enriched in the TGF-β signaling pathway in WW sheep. Bone morphogenetic protein 6 (*BMP6*) is a member of the TGF-β superfamily and was found to be highly expressed in mammalian oocytes and granulosa cells [62,63]. Studies have found that *BMP6* is involved in primary/secondary follicle transition, dominant follicle selection, ovarian steroid production, follicular atresia, the prevention of luteinization and luteolysis [64,65,66]. In addition, mice genetically deficient in *BMP6* were characterized by a reduced ovulation rate, impaired oocyte quality and impaired embryo implantation, resulting in reduced litter size [67]. Mothers against decapentaplegic homolog 4 (*SMAD4*) is a key signal transduction molecule in the TGFβ/SMAD signaling pathway, which plays an important role in the development of mammalian follicles and the proliferation and differentiation of granulosa cells [68]. Studies have found that specifically knocking out the *SMAD4* gene in ovaries led to premature failure of mouse follicles, premature luteinization of granulosa cells and decreased fertility [69]. In addition, mice die at the embryonic stage after knockout of *SMAD4* [70]. The Inhibin subunit beta B (*INHBB)* gene codes for the INHBB protein, which is secreted by ovarian granulosa cells and is mutually regulated with FSH through a feedback mechanism. The expression of INHBB mRNA and protein in the mouse oviduct is cycle-dependent and elevated during estrus, demonstrating the regulation by estrogen [71]. INHBB is secreted into the oviductal fluid via the maternal body and is essential during the early development of the embryo pre- and post-implantation. In vitro fertilized embryos deficient in INHBB often do not develop normally, and even those normally implanted and early developing embryos may die during the perinatal period [72]. S-phase kinase association protein 1 (*SKP1*) is a key skeleton protein in Skp1-Cull-F-box protein (SCF), which mediates the ubiquitination and degradation of different cyclins [73], thereby promoting the cell cycle [74]. SCF has also been found to be crucial for oocyte division and maturation [75], as well as the process of fertilization and implantation [76]. Transforming growth factor beta receptor 2 (*TGFBR2*) is responsible for encoding a transmembrane glycoprotein receptor that binds to TGF-β ligands and co-transmits signals with *TGFBR1*. It has been shown that the knockdown of *TGFBR2* disables the transduction of the TGF-β signaling pathway [77], and a synonymous mutation in *TGFBR2* (g.5058476C > T) was significantly associated with litter size in Hu sheep [78]. Here, the results imply that these target genes are probably related to the sheep reproduction process, but the molecular mechanism by which they affect fecundity remains unclear. Further experiments are needed to verify these target genes.

In the comparisons between the *FecB*^BB^ genotype and *FecB*^++^ genotype, two DE miRNAs were screened in the MF vs. WF group and 16 DE miRNAs were screened in the ML vs. WL group. Remarkably, the expression of miR-148a was tens to hundreds of times higher in the ML vs. WL group relative to other DE miRNAs, and the expression was higher in WW sheep than MM sheep during the luteal phase. It was found that miR-148a inhibited myoblast proliferation via post-transcriptional down-regulation of *KLF6* levels [79]. Moreover, miR-148a was able to regulate the placental genome, affected the development of the placenta and influenced the immune process [80], and was also associated with preeclampsia (gestational hypertension) [81]. In summary, miR-148a may have an important role in sheep oviductal reproduction, and the specific mechanism remains to be investigated.

### 4.4. Regulatory Networks of miRNAs and circRNAs

The binding relationship between circRNAs and miRNAs was predicted by the miRNA target gene prediction method. The results showed that 153 miRNAs may bind to 2824 circRNAs. Twenty-seven miRNAs were found to potentially bind to 13 DE circRNAs in MF vs. ML. circRNAs can act as a sponge to adsorb miRNAs, so we focused on circRNAs–miRNAs with opposite expression trends. In this study, we identified several key circRNA–miRNA pairs. This study reveals that miR-22-3p (target to novel_circ_0004065) was differentially expressed between pregnant and non-pregnant sheep groups and may be a key miRNA affecting implantation in early pregnancy [82]. Abnormal expression of miR-127 (target to novel_circ_0000417) may cause developmental defects in transgenic cloned sheep [83]. In addition, miR-136 (target to novel_circ_0000417) can be adsorbed by hsa_circ_0118530 and affect the granulosa cell apoptotic process [84]. miR-136 was involved in the down-regulation of luteinizing hormone receptor (*LHR*) mRNA through direct binding to LHR mRNA [85]. This study shows that miR-27a (target to novel_circ_ 0014274) bound to Ubiquitin-specific protease 25 (USP25) and thus inhibited the migration and invasion of trophoblast cells [86]. miR-27a inhibits the proliferation and promotes the apoptosis of mouse granulosa cells by reducing key enzymes for estrogen synthesis [87]. The sequencing of follicular and luteal phase ovarian tissues from monotocous and polytocous sheep revealed that oar-miR-181a (target to novel_circ_0017815) and oar-miR-27a have a targeting relationship with circLTBP1 [88]. miR-181a was also found to be associated with the regulation of estradiol synthesis and follicular apoptosis [89,90]. This may indicate that the relevant circRNAs may influence the reproductive process in sheep by binding miRNAs. Given that these circRNAs contain miRNA binding sites, they can be the subject of subsequent studies on the interactions between circRNAs and miRNAs.

## 5. Conclusions

In this study, we established the circRNA and miRNA expression profile in the oviduct during the follicular phase and luteal phase of sheep with two *FecB* genotypes. Fifteen DE circRNAs were identified in MF vs. ML. Enrichment analysis identified three DE circRNAs associated with the reproductive process in sheep. A total of 29 and 11 DE miRNAs were identified in MF vs. ML and WF vs. WL; two and 16 DE miRNAs were identified in MF vs. WF and ML vs. WL. Enrichment analysis showed that the target genes of some DE miRNAs were enriched in insulin secretion, the cAMP signaling pathway, the cGMP–PKG signaling pathway, the Rap1 signaling pathway, the TGF-β signaling pathway and other pathways related to reproduction. Twenty-seven miRNAs were revealed to possibly bind to 13 DE circRNAs. Our study, although not establishing direct causal relationships of the circRNA and miRNA changes, provides a valuable resource for the biology of circRNAs and miRNAs as well as contributes to further studies of reproductive processes in sheep.

## Figures and Tables

**Table 1:** Primer sequences and expected product sizes of circRNAs and miRNAs for RT-qPCR.

Gene Name	Primer Sequence (5′–3′)	Product Size (bp)
novel_circ_0000417	F: GAGCAAACTCGCTTCTGCATT	226
	R: CTCTCTCTGCGGTTTTGTGGT	
novel_circ_0000962	F: TGTCAAGCAGTGGGTTAATCAGA	152
	R: CATTAACGGCTGGTCTGGGT	
novel_circ_0005109	F: AGAACTGAGAAAAAGGTCAGACT	111
	R: CATACATTCTGGCGCTCGTG	
novel_circ_0016586	F: ATCAGAGAAGCCACCAGGAAC	128
	R: GCTTCTTACAGAGCTGGGGTC	
novel_circ_0017815	F: AAACGATGAAGATGCTGAGCC	134
	R: GCTCCCGAGGTTTGATGTCC	
β-Actin	F: CCAACCGTGAGAAGATGACC	97
	R: CCCGAGGCGTACAGGGACAG	
oar-miR-10b	F: GCGCACCCTGTAGAACCGAATTTGTG	22
oar-miR-376b-3p	F: GCGCGCATCATAGAGGAAAATCCATGT	21
oar-miR-376c-3p	F: GCGCGAACATAGAGGAAATTCCACGT	21
oar-miR-543-3p	F: GCGAAACATTCGCGGTGCACTTCTTT	23
oar-miR-665-3p	F: ACCAGTAGGCCGAGGCCC	22
oar-miR-U6	F: CAAGGATGACACGCAAATTCG	21

**Table 2:** Summary of the oviductal circRNA sequencing data.

Sample Name	Raw Reads	Clean Reads	Total Mapped	Q30 (%)	Aligned Rate (%)	GC Content (%)
MM_F_O_1	96,196,362	94,744,878	87,141,547	94.86	91.97	47.56
MM_F_O_2	92,640,970	90,020,528	82,821,574	93.89	92	48.67
MM_F_O_3	93,009,358	90,394,288	80,814,953	93.88	89.4	50.38
MM_L_O_1	88,588,260	85,274,304	74,294,998	92.5	87.12	47.62
MM_L_O_2	97,001,262	93,030,528	83,370,890	92.82	89.62	48.06
MM_L_O_3	96,027,084	92,170,182	78,243,273	92.77	84.89	50.11
ww_F_O_1	90,181,468	87,668,706	79,148,368	94.1	90.28	49.15
ww_F_O_2	109,389,164	106,239,400	94,022,401	94.19	88.5	50.44
ww_F_O_3	84,735,812	81,446,054	73,026,516	93.15	89.66	48.51
ww_L_O_1	102,279,454	100,207,792	92,162,400	93.47	91.97	49.53
ww_L_O_2	94,330,442	90,209,692	81,346,141	93.23	90.17	48.46
ww_L_O_3	102,550,798	99,314,822	89,937,364	93.03	90.56	48.39

**Table 3:** Summary of the oviductal miRNA sequencing data.

Sample Name	Raw Reads	Clean Reads	Screened Reads	Total Mapped	Q30 (%)	Aligned Rate (%)
MM_F_O_1	14,546,606	14,321,558	13,569,930	12,570,515	96.53	92.64
MM_F_O_2	13,014,433	12,763,844	11,630,660	10,792,527	96.42	92.79
MM_F_O_3	12,371,162	12,148,054	11,091,183	9,918,242	94.81	89.42
MM_L_O_1	12,625,788	12,431,487	11,385,564	10,489,170	94.23	92.13
MM_L_O_2	11,973,021	11,819,020	11,119,789	10,130,283	94.02	91.10
MM_L_O_3	10,296,511	10,176,943	9,946,122	8,720,780	95.77	87.68
ww_F_O_1	12,163,978	11,962,622	10,823,047	9,705,421	94.68	89.67
ww_F_O_2	11,447,533	11,252,977	10,467,663	9,283,625	94.84	88.69
ww_F_O_3	13,365,195	13,090,842	11,603,455	10,748,779	93.56	92.63
ww_L_O_1	12,185,470	11,826,433	11,381,948	10,275,416	95.50	90.28
ww_L_O_2	11,800,083	11,594,302	11,237,422	10,177,899	95.70	90.57
ww_L_O_3	11,410,997	11,179,464	10,446,590	9,313,721	95.39	89.16

## Data Availability

All the data obtained from RNA sequencing have been deposited in the Sequence Read Archive databases (The accession number of BioProject is PRJNA658731).

## Supplementary Materials

The following are available online at https://www.mdpi.com/article/10.3390/ani11102826/s1, Table S1: The basic information of circRNAs and chromosomes, Table S2: Sequence of known miRNAs and novel miRNAs, Table S3: List of DE circRNAs, Table S4: List of DE miRNAs, Table S5: Prediction of target relationship of circRNAs and miRNAs. Figure S1: Heatmap of DE miRNAs.
