# Supplementary figures and images for: Analysis of Expression Profiles of CircRNA and MiRNA in Oviduct during the Follicular and Luteal Phases of Sheep with Two Fecundity (FecB Gene) Genotypes
Source: Animals (Basel). 2021 Sep 28;11(10):2826. doi: 10.3390/ani11102826 (PMC8532869; doi:10.3390/ani11102826)

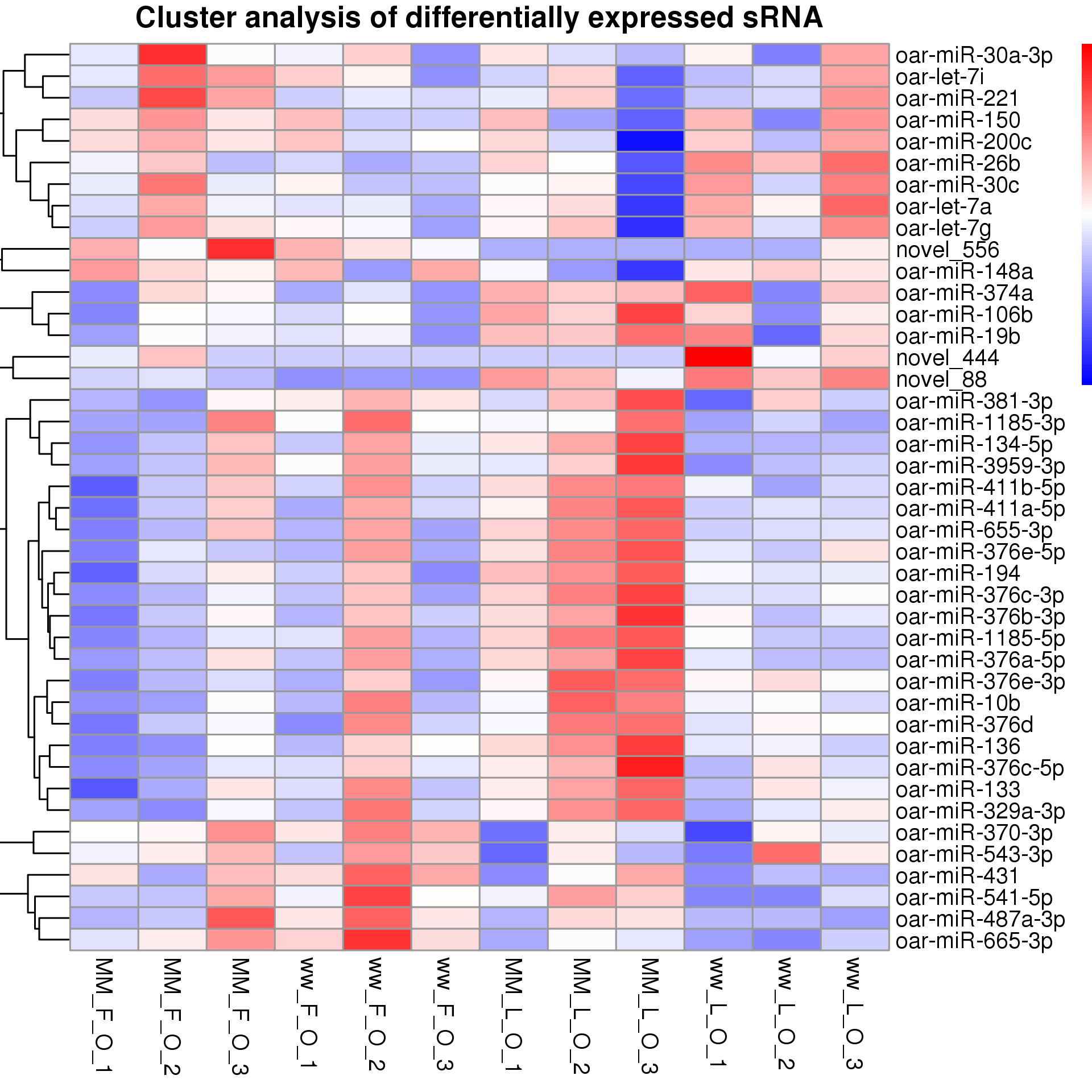

Supplement: Supplementary file 1 [file animals-11-02826-s001.zip › S1.png]
